# Dr. Shortridge’s Case of Empyema Terminating Favourably by Spontaneous Opening

**Published:** 1842-11-12

**Authors:** Samuel Shortridge

**Affiliations:** Surgeon, Port-Glasgow


### ANALECTA.

*Case of Empyema terminating favourably by spontaneous opening.* By Samuel Shortridge, Esq., Surgeon, Port-Glasgow.—Upon 5th October, 1838, I was taken to visit J. P., of a stout robust appearance, who presented the usual symptoms of acute inflammatory fever, accompanied with cough, dyspnœa, and a fixed pain near to the inferior border of the left scapula, extending across the posterior surface of the chest.

He mentioned that, two days previously, while engaged in raising a heavy piece of timber, "he felt something give a crack" in the foregoing position, which, however, did not prevent him continuing his employment during the entire day, but that, upon the following day, while rowing a boat, he became chilly and squeamish, and was forced to bed, when the above symptoms made their appearance. When seen by me, I took blood to syncope, and prescribed an antimonial saline mixture, some powders containing calomel and antimonial powder, and applied a sinapism to the painful part.

On the following day, there being no improvement, the blood-letting was repeated, a large blister was applied, and the medicine continued. The sputa, which now became more copious, from its rusty character, and the ordinary stethoscopic indications, enabled me to consider the inflammatory action to have extended to the substance of the lung. The usual antiphlogistic measures were continued; blisters repeated from time to time; and in the course of a few days, the acute febrile symptoms subsided; but there still remained the fixed pain near the scapula, frequent cough, copious expectoration, and a total inability to lie upon the right side, any attempt at which greatly aggravated the cough and dyspnœa. Very little benefit was derived from the treatment adopted; colliquative perspirations were superadded, to ameliorate which, mineral acid and quinine were had recourse to.

Towards the end of December, debility and emaciation had advanced progressively, and a fullness was observed over the left side of the chest, although no difference in its capacity was found upon measurement; percussion on the part elicited a dull sound; and the respiratory murmur was very faint, although audible over the subclavian region; and a few days after, a soft tumour appeared on the fifth intercostal space, an inch below, and to the outside of the left nipple. Upon the day of this discovery I contemplated the porpriety of puncturing the tumour, but deferred doing so till the following day, requesting support to be given by a bandage, lest it might be ruptured during coughing; as I had some expectation that the cavity might be ruptured, since, during the few days previously, there had taken place an ejection of purulent fluid, occasionally to the extent of two pints at one time, which flowed freely, and without effort, from the mouth, and sometimes so as to endanger suffocation.

Having delayed the puncture, as hinted, I found the next day that the surface of the tumour had ulcerated, and my patient lay bathed in purulent matter, the bed and clothes being fully saturated with it. The purulent matter continued to flow from the aperture, and some came afterwards by the mouth. My patient was afterwards kept comfortable by evacuating the purulent matter night and morning to the extent of a pint, which was facilitated by turning him to the left side, and requesting a full inspiration and a cough to be made, when a soft pledget with a quantity of tow and a bandage was applied.

The cough and dyspnœa were now greatly relieved; a generous diet with porter, together with the acid and quinine, were prescribed, under which he gradually gained so much strength as allowed him to go out of doors by March, 1839, and henceforward he continued still so weakly as only to be able to walk a very short distance, while the purulent matter was continued to be evacuated night and morning, in nearly the same quantities as at first, by removing the bandages, &c. until three weeks ago, when blood appeared instead of purulent matter, and the wound thereafter closed, leaving a flattened thimble-like cavity, into which the point of the finger could be introduced; and he has since progressed in strength.

Upon examination, the movements of both sides of the chest are equable, and the respiratory murmur natural, as also the sound elicited by percussion.

The successful result of this case may, perhaps, be regarded as a support to the practice generally adopted, of gradually evacuating such collections, as it is very probable that only as much purulent matter flowed in the first instance as reduced it to the level of the aperture.

Whether or not the *origo mali* was some injury the pleura could have suffered while making the vigorous effort stated, or was the result of the chill while in the boat, or both combined, may appear doubtful, but that the former had some considerable share, may be argued from the pain persisting in that locality, even up to within a short period of his final recovery.*—*Edinburgh Med. and Surg. Journ.* Oct. 1, 1842.

* The case here detailed by Mr. Shortridge is extremely important, in illustrating the mode which nature often adopts, in order to effect a cure in cases of pleurisy terminating in empyema. In all cases in which, as in the present, a circumscribed pointing tumour is formed, it is of little moment whether the puncture be made or not. It may be made with much greater chance of ultimate recovery, than where no such pointing tumour is formed. But if not made, the tumour forms a spontaneous opening as in the present case. The reason seems to be, that adhesions to considerable extent have previously taken place internally, and have thus circumscribed the matter, which, according to the principle long ago pointed out by John Hunter, finds its way naturally to one or other of the surfaces of the body.

When no pointing tumour forms, there is a manifest and decided contraindication to the operation; and it very rarely succeeds in such circumstances.—Editor.

